# *RYR2*, *PTDSS1* and *AREG* genes are implicated in a Lebanese population-based study of copy number variation in autism

**DOI:** 10.1038/srep19088

**Published:** 2016-01-08

**Authors:** Jihane Soueid, Silva Kourtian, Nadine J. Makhoul, Joelle Makoukji, Sariah Haddad, Simona S. Ghanem, Firas Kobeissy, Rose-Mary Boustany

**Affiliations:** 1 American University of Beirut Medical Center Special Kids Clinic, Neurogenetics Program and Division of Pediatric Neurology, Departments of Pediatrics and Adolescent Medicine, Lebanon; 2 Biochemistry and Molecular Genetics, American university of Beirut, Lebanon; 3 Department of Biological and Environmental Sciences, Faculty of Science, Beirut Arab University, Lebanon

## Abstract

Autism Spectrum Disorders (ASDs) are a group of neurodevelopmental disorders characterized by ritualistic-repetitive behaviors and impaired verbal and non-verbal communication. Objectives were to determine the contribution of genetic variation to ASDs in the Lebanese. Affymetrix Cytogenetics Whole-Genome 2.7 M and CytoScan^™^ HD Arrays were used to detect CNVs in 41 Lebanese autistic children and 35 non-autistic, developmentally delayed and intellectually disabled patients. 33 normal participants were used as controls. 16 *de novo* CNVs and 57 inherited CNVs, including recognized pathogenic 16p11.2 duplications and 2p16.3 deletions were identified. A duplication at 1q43 classified as likely pathogenic encompasses *RYR2* as a potential ASD candidate gene. This previously identified CNV has been classified as both pathogenic, and, of uncertain significance. A duplication of unknown significance at 10q11.22, proposed as a modulator for phenotypic disease expression in Rett syndrome, was also identified. The novel potential autism susceptibility genes *PTDSS1 and AREG* were uncovered and warrant further genetic and functional analyses. Previously described and novel genetic targets in ASD were identified in Lebanese families with autism. These findings may lead to improved diagnosis of ASDs and informed genetic counseling, and may also lead to untapped therapeutic targets applicable to Lebanese and non-Lebanese patients.

Autism Spectrum Disorders (ASD) are a group of non-progressive neurodevelopmental disorders characterized by repetitive/restrictive behaviors and impaired language/social behavior that set in between 16 to 36 months of age. Deficits are noted within the areas of communicative, interactive and behavioral core domains. These may be coupled with mental retardation (70%)^1^ and seizures (20–25%)^2^,^3^. Prevalence estimates of autism in the general population have gradually increased over the last 20 years. Recent figures from the Center for Disease Control and Prevention state that 1 of 68 children were diagnosed with autism in the USA in 2010^4^. In the Eastern Mediterranean region, small scale studies such as the one in Saudi Arabia states that 12.5% of patients ≤to 18 years who attended child psychiatric clinics have ASD^5^. In Oman, the prevalence is lower at 1.4 per 10,000 in children less than 14 years of age^6^. In Lebanon, formal estimates for prevalence of ASD in the greater Beirut and Mount Lebanon areas are now published^7^, and are 1/66 children.

ASD is a complex disorder with clinical variability, genetic heterogeneity and high *de novo* mutation rates that can also be impacted by epigenetic factors. The concordance rate for autism has been estimated to be 88.1% in monozygotic and 30.5% in dizygotic twin pairs^8^. Yet, several factors hinder identification of culprit gene(s)^9^,^10^,^11^,^12^. The fact that affected individuals seldom bear children, makes it difficult to perform linkage studies searching for ASD loci. A few recurrent aberrations have been reported and include maternally derived duplication of chromosome band 15q11.2-13. This was identified in 0.5–3% of ASD cases^13^. In addition, autistic features often occur in single gene disorders such as tuberous sclerosis, Fragile X syndrome, and Rett syndrome, but these disorders only explain around 2–5% of patients presenting with autism symptoms^14^,^15^,^16^. These disorders are no longer classified as being on the autism spectrum. Both copy number variations (CNVs) and mutations in more than 100 genes including *SHANK3*, *NLGN3*, *NLGN4*, and *NRXN1*, have been reported in rare cases of ASD^17^,^18^,^19^.

Recently, next generation sequencing in large ASD cohorts has added multiple risk-conferring genes. One of the most successful findings is the discovery of the CDH8 gene. Multiple independent studies have reported the contribution of *de novo* deleterious mutations in chromo-domain helicase DNA-binding protein 8 (CDH8) in ASD^20^,^21^,^22^,^23^. Subjects with CDH8 mutations share common features including macrocephaly, distinct facial features and gastrointestinal difficulties, which define a distinct ASD subtype^24^. Later, CDH8 was found to be involved in a highly interconnected gene network with numerous previously identified ASD candidate genes^25^.

The unique genetic makeup of the Lebanese makes them an ideal population to study and identify novel autism-related genes. The Lebanese population is genetically distinguished by significant shared ancestry^26^,^27^, a distinctive blend of DNA recombination events occurring over millennia and high rates of consanguinity^28^,^29^,^30^. The specific genetic fingerprint of the Lebanese population is ideally suited for Comparative Genomic Hybridization or CGH microarray studies, homozygosity mapping and copy number variation (CNV) analysis for discovery of novel as well as previously described ASD susceptibility genes. In fact, in the Lebanese population, male to female ASD cases are 1:1.05, unlike the reported 1:4 ratio reported elsewhere, underscoring the important contribution of unique genetic characteristics to the incidence of ASD in Lebanon^7^.

Array chromosomal genomic hybridization has increased identification of putative ASD genes, and raised up to 25% the percentage of children for whom an ASD-related genetic change can be identified^31^. CGH microarray analysis allows identification of normal benign CNVs, pathogenic CNVs associated with disease, and novel CNVs classified as unknown, but that may be associated with autism.

In this work, the objective was to determine the potential contribution of genomic variation in ASD in the Lebanese population. Affymetrix Cytogenetics Whole-Genome 2.7 M and CytoScan™ HD Arrays were used to identify CNVs across families of ASD children. We identified loci and genes that have been previously detected in the West. Additionally, we discovered new potential candidate ASD susceptibility loci and several candidate genes that code for proteins that function within the synapse. A systems biology approach was utilized to study the implications and contributions of these genes to the pathology of autism.

## Results

**Figure 1:**
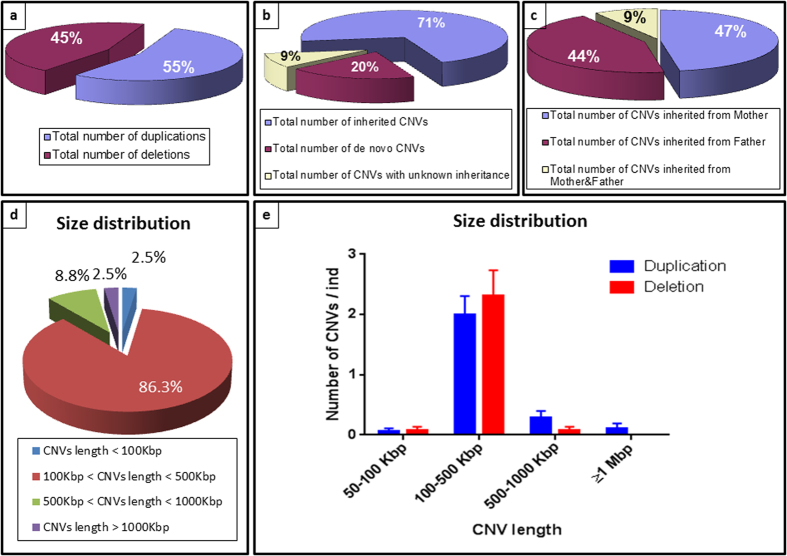
Comparative CNV landscape identified in Lebanese autism patients. Charts summarize (**a**) Number of duplications versus deletions; (**b**) Mode of inheritance of CNVs; (**c**) Number of CNVs inherited from father versus mother; (**d**) Size distribution of total CNVs; (**e**) Size distribution of deletions versus duplications.

The chromosomal microarray analysis of 41 children with autism revealed 80 CNVs (44 gains, 36 losses) (Fig. 1a). Of these, 16 CNVs are *de novo* (20%) and 57 CNVs are inherited (71%) (Fig. 1b).

2 CNVs (2.5%) were less than 100 Kbp, 69 CNVs (86.3%) ranged between 100 Kbp and 500 Kbp, 7 CNVs (8.8%) ranged between 500 Kbp and 1000 Kbp and 2 CNVs (2.5%) were above 1000 Kbp (Fig. 1d). Duplications and deletions were not distributed equally, and, deletions tended to be smaller than duplications (Fig. 1e).

A CNV (duplication at 1q43) was validated using quantitative PCR (qPCR) to ensure reliability of the CNV detection method employed (see Supplementary figure 2).

### De novo CNVs

Sixteen *de novo* CNVs (11 gains and 5 losses) were detected in 10 children with autism from 9/35 (25%) of families fully analyzed. The frequency of *de novo* CNVs in ASD patients was 27% (10 patients out of 37 analyzed cases showed at least one *de novo* CNV). The rate of *de novo* CNVs per child was 1.6 (16 *de novo* CNVs were observed in 10 patients). Table 1 and Supplementary table 1 list the 16 *de novo* CNVs and their gene content.

**Figure 2:**
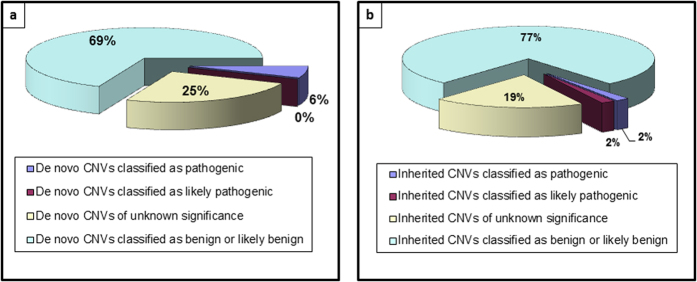
Pie charts summarizing the classification of (a) *de novo* and (b) inherited CNVs in autism patients.

We identified one duplication located on chromosome 16 (16p11.2), which is classified as pathogenic, and 4 CNVs of unclear clinical significance located on chromosomes 1, 3, 4 and 10, respectively (1q42.3, 3q24, 4q13.3, 10q26.13) (Fig. 2a).

### Inherited CNVs

57 CNVs (26 gains and 31 losses) inherited from at least one of the parents were detected in 26 of 35 families fully analyzed (two families had 2 affected children each making the total number of cases analyzed 37 out of 41). Table 2 and Supplementary table 2 list the 57 inherited CNVs and their gene content. Twenty seven out of 57 CNVs are inherited from the mother (47%) and 25 from the father (44%). Five CNVs were inherited from both the mother and father (Fig. 1c).

A deletion of 137 Kbps inherited from the father was identified. It is classified as pathogenic. It is located on 2p16.3 and encompasses the gene *NRXN1* (deletion from exon1 to exon 6). Another duplication affecting 1q43 and inherited from the mother was classified as likely pathogenic. Eleven CNVs of unclear clinical significance were also identified at 4q28.3, 5q35.2, 7q22.3, 8q21.1, 10q11.22, 14q12, 20p11.23, Xp22.33, Yp11.31 and Yp11.2 (Fig. 2b).

### Comparison of autism patients with controls, and non-autistic developmentally delayed/intellectually disabled (DD/ID) patients

**Figure 3:**
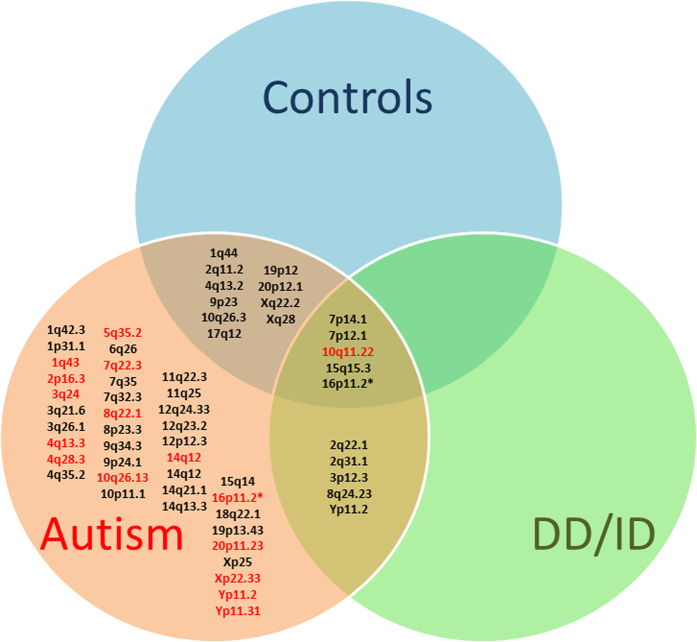
Venn diagram depicting the comparison of CNVs found in autism patients (red circle), healthy controls (blue circle), and those with developmental delay/intellectual disabilities, DD/ID (green circle). Only CNVs identified in autism patients are listed in the figure. Benign CNVs are indicated in black. Pathogenic CNVs, likely pathogenic CNVs and CNVs of unknown significance are indicated in red. *Two different CNVs are identified at 16p11.2 (pathogenic is in red and benign in black).

Some autistic patients initially diagnosed as just mentally retarded miss out on early intensive behavioral intervention, which alleviates symptoms and promotes improvement. To assess the difference at the genetic level between these two groups, we compared CNVs found in autism patients with those in the DD/ID group and in healthy controls (Fig. 3).

All the CNVs classified as pathogenic, likely pathogenic and of unknown significance were restricted to the autistic group, except for one CNV, a duplication of unknown significance at 10q11.22 which was common to all three groups (autistics, DD/ID and normal controls). This duplication of ~1000 Kbp was inherited from one of the parents in 2 different autistic families. In one of these families, we observed this duplication in the autistic boy but not in his autistic sister with mental retardation. It was also found in 3/35 patients of the DD/ID group and in 5/37 healthy controls.

The 20 CNVs that were common to the autistic group and either normal controls and/or the DD/ID groups were all classified as benign except for a duplication of unknown significance at 10q11.22 (Fig. 3). Five CNVs common to the 3 groups were located at 7p14.1, 7p12.1, 10q11.22, 15q15.3 and 16p11.2 (Table 3). Five CNVs were common to the autism group and DD/ID group only and these were located at 2q22.1, 2q31.1, 3p12.3, 8q24.23 and Yp11.2 (Table 4).

**Figure 4:**
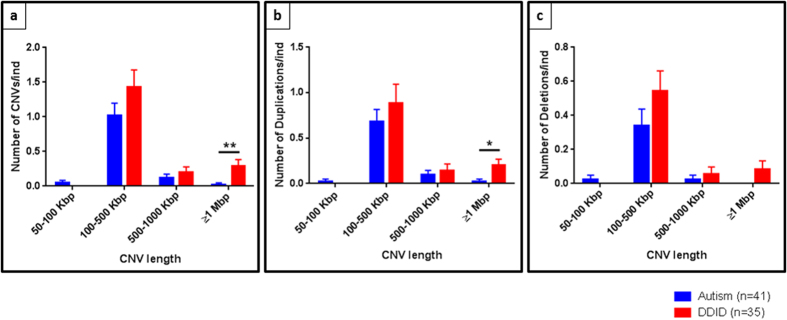
Size distribution of CNVs in autism patients (in blue) versus the Developmentally Delayed/intellectually disabled (DD/ID) group (in red). Data are means ± SD. (**a**) The DD/ID group have more CNVs per individual than autism patients except for CNVs that are smaller than 100Kbp. The trend is similar when we look at (**b**) duplications and (**c**) deletions (**p value = 0.0082 ; *p value = 0.00209; Mann-Whitney test).

After excluding all CNVs common to healthy controls and autism or DD/ID patients, we compared the length of the remaining CNVs in the autistic group versus the DD/ID group (Fig. 4). The DD/ID group has a greater number of CNVs per individual than autism patients, except for CNVs smaller than 100 Kbp which were slightly increased in ASD (0.05 in autism vs 0 in DD/ID) (Fig. 4a). This increase in CNVs in DD/ID controls is significantly more pronounced for CNVs greater than 1 Mbp (0.02 in autism vs 0.29 in DD/ID, p = 0.0082). The same trend between ASD and DD/ID was observed when duplications (Fig. 4b) or deletions (Fig. 4c) were analyzed separately. Thus, DD/ID individuals have an enrichment in total number of CNVs, especially larger CNVs (>1Mbp), whereas autism patients show an enrichment in the number of smaller deletions (<100 Kb).

### Gene content analysis

**Figure 5:**
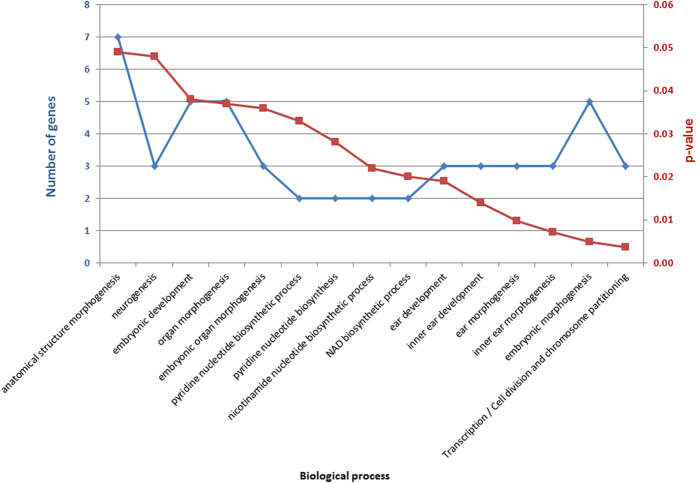
Functional categories of CNV-related genes by gene ontology analysis ranked by p-value (Corrected P values < 0.05). The second bar plot represents gene counts within each GO category.

Subsequent analysis focused on the 19 CNVs that were classified as pathogenic, likely pathogenic and of unknown significance. These CNVs were mapped across 13 chromosomes (Supplementary figure 1). Gene content was first analyzed using the Database for Annotation, Visualization and Integrated Discovery (DAVID) to assess statistical enrichment of Gene Ontology (GO) (Supplementary table 3). In order to increase significance, we selected all p-value less than 0.05. Among GO biological processes, the most significant enrichment was in transcription, cell division and chromosome partitioning (3 genes, p = 0.004) (Fig. 5).

Genes involved in cell and organ development (including embryonic development, organ morphogenesis, and neurogenesis) were also identified.

**Figure 6:**
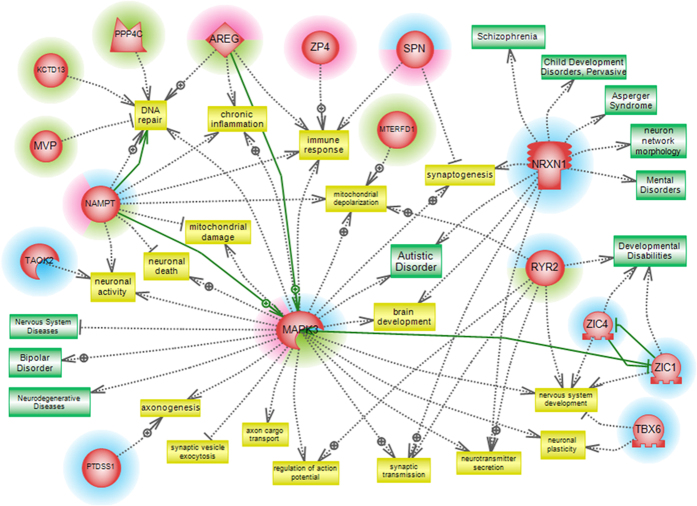
Pathway Studio analysis of 16 potential candidate genes. A biological network was created using the Pathway Studio 9.0 program to identify biological functions and disorders associated with potential candidate genes selected from pathogenic and likely pathogenic CNVs, and from CNVs of unknown significance. Out of 88 genes, 16 genes were found to be involved in direct interactions. 3 different functional sub-networks were detected in this pathway analysis: components of the first sub-network related to nervous system functions are highlighted with a blue halo; Components of the second sub-network related to immune response are highlighted with a red halo; Components of the third sub-network related to mitochondrial dysfunction and DNA repair are highlighted with a green halo. Green arrows show possible direct interactions between genes.

Using Pathway Studio software, a network was generated linking genes with selected functional biological processes and neurological diseases. The network was also used to investigate gene-gene interactions possibly existing among genes (Fig. 6). The functional processes in this network, such as neurogenesis, axonogenesis, DNA repair, mitochondrial damage and immune response where manually chosen for their relevance to identified deficits in ASDs. The network generated included 16 genes and 3 different functional sub-networks. The number of studies and citations used to draw each interaction has been included in Supplementary table 4. The first sub-network includes *SPN*, *NRXN1*, *RYR2*, *ZIC4*, *TBX6*, *MAPK3, ZIC1, PTDSS1, TAOK2* related to nervous system functions. The second sub-network includes *SPN*, *AREG*, *NAMPT, ZP4* and *MAPK3* related to the immune response. The third sub-network includes *NAMPT*, *PPP4C, MVP, KCTD13, MTERFD1, AREG* and *MAPK3* related to mitochondrial dysfunction and DNA repair. Of the 16 genes, *MAPK3* shows the highest “connectivity” with other components within the 3 sub-networks. *MAPK3* is related to autism, neurodegenerative diseases and bipolar disorder, and can exacerbate nervous system diseases. *NRXN1* is related to autism, Asperger, schizophrenia, and pervasive developmental disorder.

## Discussion

This is the first genome-wide CNV association study in 41 autism patients in the Lebanese screening the genome for microdeletions and microduplications.

Compared to healthy controls, patients show a decreased number of CNVs (1.9 CNV per individual in autistics versus 3.5 CNV per individual in normal controls; p-value = 0.0003). This finding contradicts previous studies claiming larger number of rare *de novo* CNVs^32^,^33^ and common genetic variants^34^ in autism. Here, the frequency of *de novo* CNVs in ASD patients was 27% and the rate of *de novo* CNVs per child was 1.6. This is much higher than what has been previously described (frequency of 5.5 to 10%^32^,^33^,^35^,^36^,^37^, and a rate of 1.1^35^,^36^). These discrepancies may represent specific characteristics of the Lebanese population, but may also be due to the small number of cases analyzed.

Eighty six percent (86.3%) of CNVs were 100–500 kbp in size. When comparing CNV length, duplications were enriched for the larger size (>500 kbp), while deletions were enriched for the smaller 50–500 kbp size. Our findings reproduce results of large-scale genome-wide surveys on ASD individuals of European ancestry^32^,^38^. Large CNVs lead to significant effects as they perturb dosage of many genes. Multiple small events may hit critical genes simultaneously and have an overall significant effect.

Two pathogenic CNVs (duplication 16p11.2 and deletion 2p16.3) and a likely pathogenic duplication (1q43) were identified in 2 (4.8%) of the ASD patients (Tables 1 and 2).

Two pathogenic CNVs identified in the same patient (Family LAS17) were located at previously reported ASD loci, including a *de novo* duplication at 16p11.2, and a deletion inherited from the father at 2p16.3. The region encompassing 16p11.2 (MIM 614671) is a genomic and variably expressed hotspot that is associated with neurological diseases^39^,^40^. CNVs at 16p11.2 are genetically linked to 1% of autism-related disorders^39^. This interval described by us and in the literature, contains several genes suspected to have a role in autism, especially the MAPK3 gene, which expresses the MAP kinase protein (ERK1). The 2p16.3 deletion described by us and inherited from the father implicates exons 1 to 6 of the neurexin gene (*NRXN1)*. This deletion is associated with neuropsychiatric and neurodevelopmental disorders, including ASD^36^,^41^,^42^,^43^,^44^, schizophrenia^45^,^46^, and dysmorphic features. The *NRXN1* gene is a presynaptic neuronal protein and is required for synaptic formation and efficient neurotransmission^47^.

We classified a maternally inherited duplication at 1q43 as likely pathogenic. It encompasses the genes *RYR2*, *LOC100130331* and *ZP4*. It overlaps CNVs found in 3 cases from the International Standards for Cytogenomic Arrays (ISCA) consortium with developmental delay and/or other significant developmental or morphological phenotypes^48^. One case from ISCA has a duplication (nssv578551 at 1:237418348-239615755) and is classified as pathogenic without validation. This duplication encompasses the genes *RYR2*, *LOC100130331*, *ZP4* and *CHRM3*. Two other ISCA cases bear a duplication of uncertain significance (nssv583285 at 1:237606586-23794780 and nssv1415541 at 1:237910201-237994811) that involves only the *RYR2* gene. The duplication described at 1q43 overlaps a duplication (1:237694745-237824100) within the *RYR2* gene found in a patient (DECIPHER ID. 248533) with autism, intellectual disability and an unspecified morphological phenotype. RYR2 protein is a member of the family of intracellular ryanodine receptor Ca2 +-release channels implicated in several disorders^49^,^50^. RYR2 is expressed in the brain and contributes to regulation of cytosolic calcium dynamics. A recent study reveals a SNP association highlighting *RYR2* as a potential ASD risk gene^51^. Evidence supports a role for RYR2-mediated control of calcium homeostasis in stress-induced defects in cognitive function and postsynaptic plasticity in hippocampal neurons^52^.

Also, 12/41 of patients (29%) had aberrations of unknown significance with no reports in databases or the literature. Among those CNVs a duplication was found at 10q11.22 in 2/41 in autism patients from two families (SES3 and LAS9). This duplication overlaps benign CNVs in the DGV database. In DECIPHER, this duplication overlaps CNVs found in patients with autism and/or intellectual disabilities. A similar duplication at 10q11.22 in two patients with Rett syndrome that includes two candidate modifier genes (*GPRIN2* and *PPYR1*) is reported^53^. GPRIN2 is highly expressed in the cerebellum and regulates neurite outgrowth. PPYR1, known as neuropeptide Y receptor or pancreatic polypeptide 1, is a key regulator of energy homeostasis, and is directly involved in the regulation of food intake. We found this same 10q11.22 duplication in 3/35 patients from the DD/ID group with heterogeneous phenotypes. One patient had mental retardation, the second had moderate mental retardation, language delay, stereotypies and features of autism, and the third patient had a deletion at 22q13.31q13.33 causing Phelan-McDermid syndrome (referred for mild developmental delay, dysmorphism and speech delay). This duplication was identified in 5/37 of controls, indicating this variant could be a common CNV or a pathogenic CNV with incomplete penetrance. Taken together, these facts imply that this CNV, which is common to different neurodevelopmental disorders, may modulate the phenotypic expression of disease, instead of being a single causative factor.

We performed gene ontology (GO) analysis by querying the gene content of all CNVs classified as pathogenic, likely pathogenic and of unknown significance into the records of the GO database (Fig. 5 and Supplementary table 3). Functional processes related to DNA repair, organ development, and neurogenesis, were found. Using Pathway Studio 9, functional processes impacting deficits in ASD were chosen and used to build a literature-mined network based on CNV gene content. The network generated included 16 genes of interest (Fig. 6).

Among these genes, the phosphatidylserine synthase 1 gene (*PTDSS1*) provides for a plausible ASD candidate gene and was identified in family BAK42 within a maternally inherited 152 Kbp duplication classified as ‘of unknown significance’. *PTDSS1* encodes for the phosphatidylserine synthase 1 protein that synthesizes phosphatidylserine. Phosphatidylserine is present in all membrane phospholipids in mammalian tissues and cells^54^,^55^. In addition to its roles in apoptosis, internalization of viruses and coagulation, phosphatidylserine participates in intracellular processes by interacting with key signaling proteins, including the Ras and Rho family of GTPases and protein kinase C^56^. Heterozygous missense gain-of-function mutations in *PTDSS1* were found in patients with Lenz-Majewski hyperostotic dwarfism^57^,^58^. This syndrome manifests sclerosing bone dysplasia, distinct craniofacial, dental, cutaneous, distal-limb anomalies, and intellectual deficits. Other membrane phospholipid abnormalities have been reported in many psychiatric and behavioral disorders including schizophrenia, dyslexia, and dyspraxia^59^,^60^,^61^. Membrane phospholipids, a prime target for reactive oxygen species (ROS) damage are altered in autism^62^,^63^. Phosphatidylserine levels were increased in erythrocyte membranes from children with autism compared to unaffected siblings^64^. Phosphatidylserine metabolism in the nervous system of individuals with autism may alter axonogenesis, neuronal activity, and brain development.

A second ASD candidate gene of interest generated by Pathway Studio 9 analysis is the amphiregulin gene (*AREG*), and was identified in family CLIN29 within a *de novo* 115 Kbp deletion classified as of unknown significance. Amphiregulin is a member of the epidermal growth factor family (EGF) that includes EGF, transforming growth factor-alpha (TGF-alpha), heparin-binding EGF, betacellulin and epiregulin. Amphiregulin binds to ErbB1 receptor or epidermal growth factor receptor (EGFR)^65^. Plasma EGFR levels are significantly higher in autistic children compared to neurotypic controls^66^. Amphiregulin can act as an autocrine mitogen on Schwann cells in the sciatic nerve^67^, and a survival factor for sensory neurons that stimulate axonal outgrowth via the EGF receptor^68^. Amphiregulin, similarly to FGF-2 or EGF^69^, is a mitogen for neural stem cells in culture, and promotes survival and differentiation of neuronal precursor PC12 cells^70^,^71^. Genetic studies in autism have identified mutations and copy number variations in many genes involved in the MAPK/ERK signaling pathway. A gain of function mutation in the *CACNA1C* gene, deletions and disruption of the *SYNGAP1* gene, the latter a CNV encompassing the *MAPK3* gene, all indicate that in some autism patients the ERK cascade is inappropriately activated^72^. Recent analyses clearly link the MAPK pathway to functional gene networks affected in ASDs^73^. It is proposed that *AREG* is a novel autism candidate gene, supporting the theory that alteration of MAPK/ERK signaling may be common to many autistic patients^74^.

## Conclusion

The study of Lebanese autism patients adds additional support for the contribution of known CNVs to ASD. Also, shared ancestry and high rates of consanguinity in the Lebanese led to identification of new variants and previously unrecognized susceptibility candidate genes for ASD in a relatively small number of Lebanese ASD children.

## Methods

### Subject material

We studied three different groups. Group 1 includes a total of 41 autistic children (38 boys and 3 girls). 37/41were from 35 fully analyzed families (33 with one affected and 2 with 2 affected each). The DNA from parents of 4 autistic children was not available for analysis (Supplementary table 5). Autistic children were 3–18 years of age at the time of recruitment, and fulfilled the Diagnostic and Statistical Manual of Mental Disorders V criteria for autism; group 2 includes a control cohort of 37 normal participants (19 males and 18 females); group 3 includes 35 individuals referred to our clinical neurogenetics service with non-syndromic developmental delay and intellectual disabilities (DD/ID), or rare neurological syndromes. All patients selected had a normal karyotype and were negative for fragile-X syndrome. Informed written consent was obtained in accordance with an AUBMC IRB approved protocol (ID: Bioch.RB.06), and all methods were conducted in accordance with approved guidelines and regulations.

DNA samples from all participants were extracted from peripheral blood using QIAamp® blood midi kit (Qiagen, Inc., Valencia, CA). Affymetrix Cytogenetics 2.7M arrays and CytoScan™ HD Arrays were used for CNV screening.

### Chromosomal Microarray Analysis

Chromosomal microarray analysis was performed using the Affymetrix Cytogenetics 2.7M and CytoScan™ HD arrays, according to the manufacturer’s instruction (Affymetrix Inc., Santa Clara, CA). These are chips with more than 2.6 million probes capable of detecting known and novel chromosome aberrations across the entire human genome consisting of 25 kb or larger copy number variations (CNVs). Briefly, 100–250 ng of genomic DNA is amplified, fragmented, labeled then hybridized to the arrays for 16–18 h. Arrays were then washed and stained on the GeneChip® Fluidics Station 450 and DAT images acquired using the GeneChip® Scanner 3000 7G (Affymetrix Inc.). Data analysis was performed using the Affymetrix Chromosome Analysis Suite (CHAS). Families were analyzed in trios to determine the *de novo* or inherited type of each CNV detected in the autistic patients.

### Functional classification of loci and genes

The genomic variants were evaluated using DECIPHER (http://decipher.sanger.ac.uk/), Database Genomic Variants (http://dgv.tcag.ca/dgv/app/home), and the International Collaboration for Clinical Genomics-International Standards Cytogenomic Arrays Database Search (https://www.iscaconsortium.org/) and classified into five groups: pathogenic, likely pathogenic, unknown significance, likely benign and benign. CNVs that were clearly found related to Autism in databases were classified as pathogenic. CNVs reported in several autism case studies without direct evidence of pathogenicity were classified as likely pathogenic. CNVs with no reported information, or incomplete and/or contradictory information were classified as of unknown significance. CNVs reported in databases at high frequency in control data were classified as benign or likely benign according to the degree of uncertainty.

The genes in CNV regions were classified according to biological processes using the Database for Annotation, Visualization and Integrated Discovery (DAVID) v6.7 b (http://david.abcc.ncifcrf.gov/)^75^,^76^. Gene ontology options (GOTERM_BP_ALL, GOTERM_CC_ALL, GOTERM_MF_ALL), and functional categories (COG_ONTOLOGY, and SP_PIR_KEYWORDS) were selected and a functional annotation chart generated. A maximum p-value of 0.05 was chosen to select only significant categories. For gene-enrichment analysis DAVID uses the “Ease score statistics”, an alternative name of Fisher Exact statistics, referring to the one-tail Fisher Exact test. P-values were corrected by the Benjamini correction which controls the False Discovery Rate (FDR). Network analysis was performed by Pathway Studio software version 9.0 (Ariadne Genomics, Rockville, Md., USA).

### Quantitative real-time PCR validation

Quantitative real-time PCR reactions were performed in triplicate in 96-well plates using specific primers (TIB MOLBIOL) and the iQTM SYBR® Green Supermix (BioRad) as a fluorescent detection dye, in CFX96TM Real-Time PCR (BioRad), in a final volume of 12.5 μl. The input amount of genomic DNA used in each well was 35 ng. To characterize generated amplicons and to control contamination by unspecific by-products, melt curve analysis is applied. All results were normalized to *GAPDH* level and calculated using the ΔΔC_T_ method. Primer sequences are *GAPDH* Fwd: CGAGATCCCTCCAAAATCAA; *GAPDH* Rev: AGGCATTGCTGCAAAGAAAG; *RYR2* Fwd: TGCGTGCTGGCTACTATGAC; *RYR2* Rev: TGCTTCAAGTCCTCGTTGTG.

## Additional Information

**How to cite this article**: Soueid, J. *et al.* *RYR2, PTDSS1* and *AREG* genes are implicated in a Lebanese population-based study of copy number variation in autism. *Sci. Rep.* **6**, 19088; doi: 10.1038/srep19088 (2016).

## Supplementary Material

Supplementary Information

## Figures and Tables

**Table 1:** *De novo* CNVs identified in patients with ASD classified as pathogenic, likely pathogenic or of unknown significance.

ID	Cytoband	Type	Location	Size (kb)	Genes	Classification
LAS17	16p11.2	Gain	29396001-30190676	795	YPEL3, PRRT2, SULT1A4, GDPD3, C16orf53, LOC440356, BOLA2, PPP4C, ZG16, CDIPT, HIRIP3, LOC388242, TAOK2, ASPHD1, KCTD13, INO80E, SPN, QPRT, LOC606724, SEZ6L2, MAZ, TMEM219, LOC440354, ALDOA, C16orf54, KIF22, C16orf92, SLC7A5P1, TBX6, MAPK3, MVP	Pathogenic
CLIN19	1q42.3	Gain	236270756-236557162	286	ERO1LB, GPR137B	Unknown significance
	3q24	Gain	147147614-147082426	67	ZIC1, ZIC4	Unknown significance
CLIN27	10q26.13	Gain	124808072-124919262	111	HMX3, HMX2, BUB3	Unknown significance
CLIN29	4q13.3	Loss	75405778-75521036	115	AREG	Unknown significance

**Table 2:** Inherited CNVs identified in patients with ASD classified as pathogenic, likely pathogenic or of unknown significance.

ID	Cytoband	Type	Location	Size (kb)	Genes	Classification	Inheritance
SES3	10q11.22	Gain	46776777-47939116	1162	GPRIN2, FAM35B2, ANTXRL, FAM35B, SYT15, AGAP9, ANXA8L2, PPYR1, FAM21B, FAM25C, LOC642826, ANXA8, LOC643650	Unknown significance	Mother
LAS9	10q11.22	Gain	46950168-47939116	989	FAM21B/25B/35B2, SYT15, ANXA8L2, ANTXRL, PPYR1, AGAP9, LOC643650/642826, GPRIN2, FAM25G	Unknown significance	Father
	7q22.3	Gain	105890353-106064794	174	NAMPT	Unknown significance	Father & Mother
LAS17	2p16.3	Loss	51166006-51302668	137	NRXN1	Pathogenic	Father
CLIN22	14q12	Gain	31642542-31778371	136	HECTD1, HEATR5A	Unknown significance	Mother
CLIN24	20p11.23	Loss	20441027-20573017	132	RALGAPA2	Unknown significance	Mother
BAK41	Yp11.31	Gain	2867178-3222952	356	LINC00278	Unknown significance	Father
	Yp11.2	Gain	3973124-4271373	298	No genes	Unknown significance	Father
BAK42	4q28.3	Loss	138092729-138196101	103	No genes	Unknown significance	Mother
	5q35.2	Loss	175438045-175638617	201	FAM153B, LOC100507387, LOC643201	Unknown significance	Father
	8q22.1	Gain	97151500-97303937	152	GDF6, MTERFD1, PTDSS1, UQCRB	Unknown significance	Mother
BAK45	1q43	Gain	237517711-238090569	573	RYR2, LOC100130331, ZP4	Likely pathogenic	Mother
	Xp22.33	Gain	1841391-2303322	462	DHRSX	Unknown significance	Father

**Table 3:** Common CNVs identified in patients with ASD, developmental and/or intellectual delays, and in healthy controls.

Cytoband	Type	Location	Size (kb)	Genes	Classification
7p14.1	Loss	38290298-38402607	112	TARP	Benign
7p12.1	Loss	53459876-53591589	132	No genes	Benign
**10q11.22**	**Gain**	**46776777-47939116**	**1162**	**GPRIN2, FAM35B2, ANTXRL, FAM35B, SYT15, AGAP9, ANXA8L2, PPYR1, FAM21B, FAM25C, LOC642826, ANXA8, LOC643650**	**Unknown significance**
15q15.3	Gain	43859057-44041632	183	CKMT1B, CATSPER2, CKMT1A, PPIP5K1, PDIA3, STRC	Benign
16p11.2	Gain	34449594-34755816	306	LOC283914, LOC146481, LOC100130700	Benign

**Table 4:** Common CNVs identified in patients with ASD and in patients with developmental and/or intellectual delays.

Cytoband	Type	Location	Size (kb)	Genes	Classification
2q22.1	Loss	142050109-142166828	117	LRP1B	Benign
2q31.1	Gain	176919432-177066433	147	HOXD3/4/8/9/10/11/12/13, EVX2	Benign
3p12.3	Gain	75093330-76311299	1218	ZNF717, FAM86D, FRG2C	Likely benign
8q24.23	Loss	137687247-137863344	176	no genes	Benign
Yp11.2	Gain	9198721-9319924	121	FAM197Y2P, TSPY1, TSPY3, TSPY4	Benign
