# Supplementary material for: RYR2, PTDSS1 and AREG genes are implicated in a Lebanese population-based study of copy number variation in autism
Source: Sci Rep. 2016 Jan 8;6:19088. doi: 10.1038/srep19088 (PMC4705475; doi:10.1038/srep19088)
Supplement: Supplementary Information [file srep19088-s1.pdf]

RYR2, *PTDSS1* and *AREG* genes are implicated in a Lebanese population-based study of copy number variation in autism

Jihane Soueid[1,*], Silva Kourtian[1,*], Nadine J Makhoul[1], Joelle Makoukji[1], Sariah Haddad[1], Simona S Ghanem[1], Firas Kobeissy[2] and Rose-Mary Boustany[1, 2 ¶]

Supplementary Figures

Supplementary Figure1.

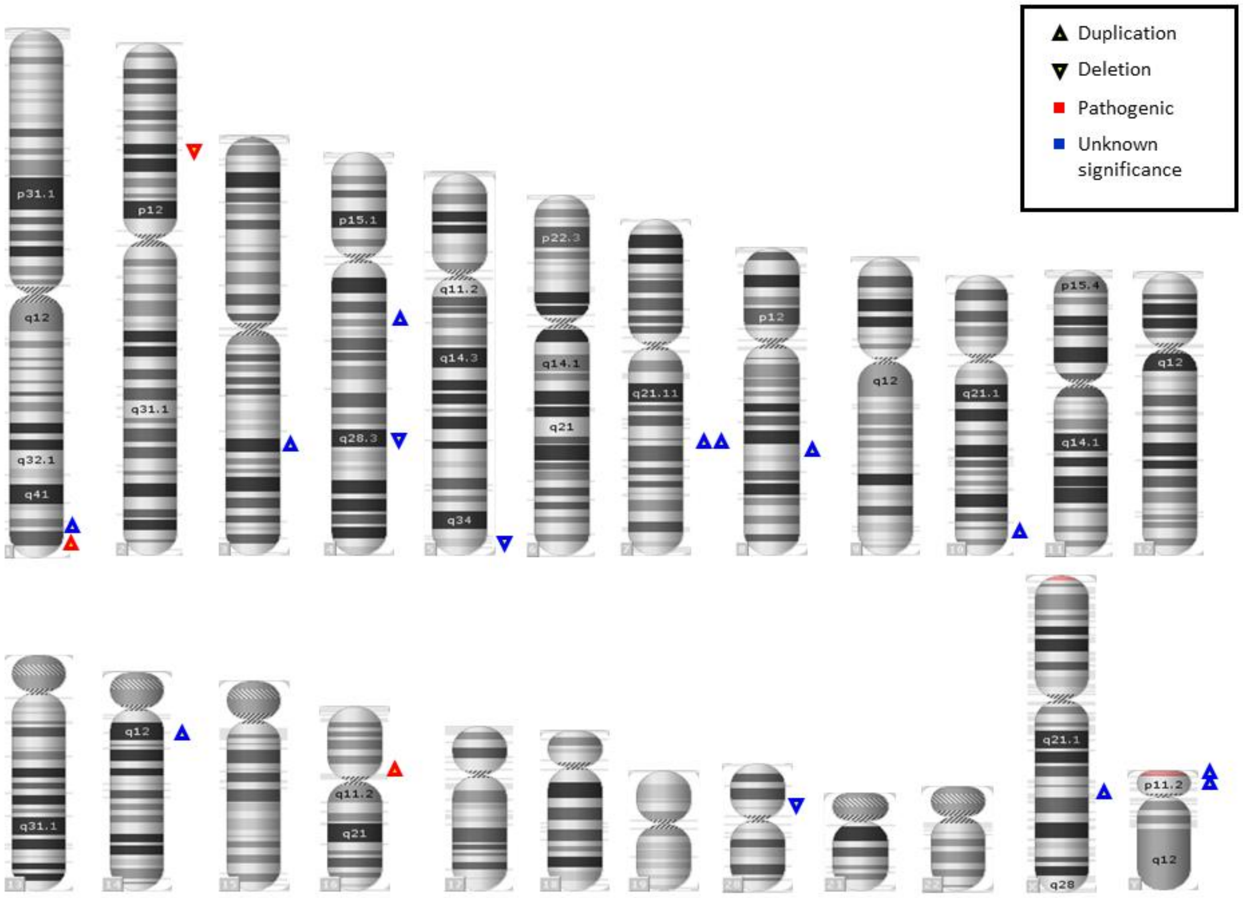

Supplementary Figure2.

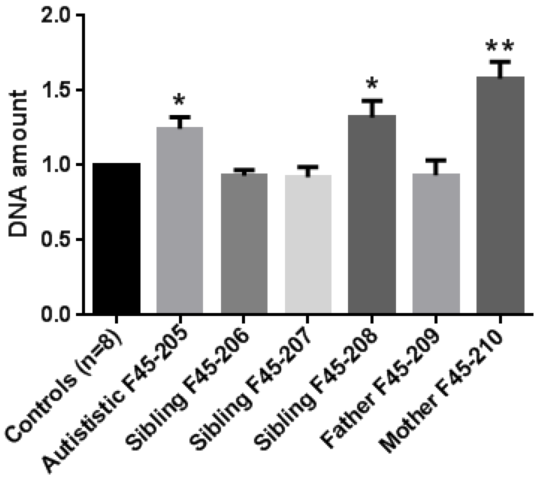

Supplementary figures legends

Supplementary figure 1. Schematic diagram portraying pathogenic CNVs (red) and CNVs of unknown significance (blue) found in Lebanese autism patients on different chromosomal positions.

Supplementary figure 2. qPCR assays to validate 1q42 duplication using primers within the exon 37 of *RYR2* gene. Values are means of the fold changes normalized to *GAPDH* DNA amount, with their standard errors represented by vertical bars.*p<0.05, **p<0.01 by Student's t-test (n=4). The healthy siblings F45-206, F45-207 and the father F45-209 have a normal copy number similar to controls, whereas the autistic patient F45-205, the healthy sibling F45-208 and the mother F45-210 show a duplication.

Supplementary tables

Supplementary table 1. *De novo* CNVs identified in patients with ASD classified as benign and likely benign.

ID	Cytoband	Type	Location	Size (kb)	Genes	Classification
LAS6	4q35.2	Gain	189335700-189519024	183	LOC401164	Likely benign
	12p12.3	Gain	19468069-19580378	112	PLEKHA5	Benign
LAS17	Yp11.2	Gain	9198721-9319924	121	FAM197Y2P, TSPY1, TSPY3, TSPY4	Benign
CLIN19	1q42.3	Gain	235164385-235611093	447	GGPS1, B3GALNT2, TOMM20, SNORA14B, ARID4B, RBM34, TBCE	Benign
CLIN24	1q44	Loss	248688571-248795277	107	OR2T10, OR2T29, OR2T34, OR2T11	Benign
	10q26.3	Gain	135252897-135372601	120	SYCE1, SPRNP1, CYP2E1, LOC619207	Benign
CLIN34	4q13.2	Gain	69367092-69540427	173	UGT2B15, UGT2B17	Benign
BAK40	3p12.3	Gain	75671971-75908054	236	ZNF717, FRG2C	Benign
	7p14.1	Loss	38290298-38402607	112	TARP	Benign
	8p23.3	Gain	684607-1219586	535	No genes	Benign
BAK42	9q34.3	Loss	140612478-140682881	70	EHMT1	Likely benign

Supplementary table 2. Inherited CNVs identified in patients with ASD classified as benign and likely benign.

ID	Cytoband	Type	Location	Size (kb)	Genes	Classification	Inheritance
SES2	7q32.3	Gain	131430119-132076332	646	PLXNA4	Likely benign	Mother
LAS6	15q15.2	Loss	43871160-43972150	101	CATSPER2, CKMT1B, STRC, PPIP5K1	Benign	Father & Mother
LAS7	9p24.1	Gain	5812163-5987927	176	MLANA, KIAA2026, ERMP1	Benign	Mother
	10p11.1	Gain	38244992-38647577	403	ZNF37A, ZNF33A, LOC100129055, ZNF25	Likely benign	Mother
	19q13.43	Gain	56310321-56517347	207	NLRP4/5/8/11/13	Benign	Mother
LAS11	9p23	Loss	11994297-12124344	130	No genes	Likely benign	Mother

LAS15	3q21.6	Gain	165203570-165406756	203	no genes	Benign	Father
	18q22.1	Loss	64070273-64294890	225	CDH19	Benign	Mother
LAS17	14q12	Loss	27537145-27737791	201	No genes	Likely benign	Father
CLIN18	2q22.1	Loss	142050109-142166828	117	LRP1B	Benign	Father
	7p12.1	Loss	53459876-53591589	132	No genes	Benign	Mother
CLIN19	2q31.1	Gain	176919432-177066433	147	HOXD3/4/8/9/10/11/12/13, EVX2	Benign	Father & Mother
	7q35	Loss	146099648-146330586	231	CNTNAP2 (intronic)	Likely benign	Mother
	15q15.3	Gain	43859057-44041632	183	CKMT1B, CATSPER2, CKMT1A, PPIP5K1, PDIA3, STRC	Benign	Father & Mother
CLIN24	22q11.23q12.1	Loss	25656237-25925078	269	CRYBB2P1, LRP5L, IGLL3P	Benign	Father
CLIN27	8q24.23	Loss	137687247-137863344	176	no genes	Benign	Mother
	14q21.1	Loss	40717147-40896831	180	No genes	Benign	Mother
SAI31	3q26.1	Loss	162182181-163158948	977	LOC647107	Likely benign	Mother
	4p15.1	Loss	28739163-28866444	127	No genes	Likely benign	Mother
SAI33	16p12.3	Gain	16293540-16857531	564	ABCC6, PKD1P1, NOMO3	Likely benign	Father
	16p12.3	Gain	18236096-18696746	461	NOMO2, ABCC6P1	Likely benign	Father
CLIN35	Xq28	Gain	148873358-149006650	133	No genes	Benign	Mother
BAK39	1p31.1	Gain	75142005-75580191	438	CRYZ, TYW3	Likely benign	Mother
	20p12.1	Loss	14788354-15093936	306	MACROD2	Benign	Father
SAI36	2q11.2	Loss	97936901-98065927	129	No genes	Likely benign	Father
	3p12.3	Gain	75093330-76311299	1218	ZNF717, FAM86D, FRG2C	Likely benign	Father
	11q25	Gain	134412773-134744182	331	no genes	Benign	Mother
BAK37	7p12.1	Loss	53460807-53591589	131	No genes	Benign	Father
BAK38	11q22.3	Loss	109067949-109181323	113	No genes	Likely benign	Mother
	12q23.2	Gain	102286925-102629437	343	C12orf48, DRAM1, CCDC53, NUP37, PMCH	Likely benign	Mother
BAK41	7p12.1	Loss	53458839-53590535	132	No genes	Benign	Mother

	10q26.3	Gain	135252346-135372601	120	LOC619207, CYP2E1, SYCE1	Benign	Father
	12q24.33	Gain	132594436-132955402	361	EP400NL, DDX51, NOC4L, GALNT9	Likely benign	Father
	17q12	Loss	36283806-36404136	120	TBC1D3, LOC440434	Likely benign	Father
BAK42	19p12	Loss	20596105-20716140	120	ZNF826P	Benign	Father
	Xq25	Loss	126889708-127147105	257	No genes	Likely benign	Mother
BAK43	9p23	Loss	12000099-12127088	127	no genes	Benign	Father
	16p11.2	Gain	34449594-34755816	306	LOC283914, LOC146481, LOC100130700	Benign	Father
	19p12	Loss	20598429-20716153	118	ZNF826P	Benign	Father
	1q44	Loss	248688586-248795277	107	OR2T10/11/29/34	Benign	Mother
BAK44	1q44	Loss	20596105-20716140	120	ZNF826P	Benign	father
	2q22.1	Loss	126889708-127147105	257	No genes	Likely benign	Mother
BAK45	19p12	Loss	20596105-20716376	120	ZNF826P	Benign	Father & Mother
BAK46	19p12	Loss	20598429-20716376	118	ZNF826P	Benign	Father

Supplementary table 3. Gene ontology (GO) enrichment analysis among genes included in all CNVs classified as pathogenic, likely pathogenic, and of unknown significance.

Category	Term	Genes	Nb of genes	Corrected P-values*
GOTERM_BP_ALL	anatomical structure morphogenesis	HMX3, HMX2, HECTD1, TBX6, ZIC1, NRXN1, SPN	7	0.049
SP_PIR_KEYWORDS	neurogenesis	HMX3, HMX2, ZIC1	3	0.048
GOTERM_BP_ALL	embryonic development	HMX3, HMX2, HECTD1, TBX6, ZIC1	5	0.038
GOTERM_BP_ALL	organ morphogenesis	HMX3, HMX2, HECTD1, TBX6, ZIC1	5	0.037
GOTERM_BP_ALL	embryonic organ morphogenesis	HMX3, HMX2, ZIC1	3	0.036
GOTERM_BP_ALL	pyridine nucleotide biosynthetic process	NAMPT, QPRT	2	0.033
SP_PIR_KEYWORDS	pyridine nucleotide biosynthesis	NAMPT, QPRT	2	0.028

GOTERM_BP_ALL	nicotinamide nucleotide biosynthetic process	NAMPT, QPRT	2	0.022
GOTERM_BP_ALL	NAD biosynthetic process	NAMPT, QPRT	2	0.020
GOTERM_BP_ALL	ear development	HMX3, HMX2, ZIC1	3	0.019
GOTERM_BP_ALL	inner ear development	HMX3, HMX2, ZIC1	3	0.014
GOTERM_BP_ALL	ear morphogenesis	HMX3, HMX2, ZIC1	3	0.010
GOTERM_BP_ALL	inner ear morphogenesis	HMX3, HMX2, ZIC1	3	0.007
GOTERM_BP_ALL	embryonic morphogenesis	HMX3, HMX2, HECTD1, TBX6, ZIC1	5	0.005
COG_ONTOLOGY	Transcription / Cell division and chromosome partitioning	MAZ, ZIC1, ZIC4	3	0.004

***Fisher Exact P-values corrected for multiple comparisons in Benjamini-Hochberg test.**

Supplementary table 4. Studies and citations for each relation within the network build between genes included in CNVs classified as pathogenic, likely pathogenic, and of unknown significance and different entities related to identified deficits in ASDs using Pathway studio.

Relation	Type	TextRef
AREG --+> DNA repair	Regulation	info:pmid/21665306#body:86
AREG --+> MAPK3	Regulation	info:pmid/23108309#abs:10
AREG --+> MAPK3	Regulation	info:pmid/16261397#abs:8
AREG --+> MAPK3	Regulation	info:pmid/12869389#abs:9
AREG --+> MAPK3	Regulation	info:pmid/12055597#abs:5
AREG --+> MAPK3	Regulation	info:pmid/17536007#abs:8
AREG --+> MAPK3	Regulation	info:pmid/12589827#abs:5
AREG --+> MAPK3	Regulation	info:pmid/21047912#abs:10
AREG --+> MAPK3	Regulation	info:pmid/20826789#abs:7
AREG --+> MAPK3	Regulation	info:pmid/21665306#body:47
AREG --+> MAPK3	Regulation	info:pmid/16314835#body:67
AREG --+> MAPK3	Regulation	info:pmid/24124521#cont:233
AREG --+> MAPK3	Regulation	info:pmid/20610537#cont:179
AREG --+> MAPK3	Regulation	info:pmid/17901238#body:189
AREG --+> MAPK3	Regulation	info:pmid/19880654#body:193
AREG --+> MAPK3	Regulation	info:pmid/23010081#body:115
AREG --+> MAPK3	Regulation	info:pmid/16543407#body:164
AREG --+> MAPK3	Regulation	info:pmid/17363371#body:195
AREG --+> MAPK3	Regulation	info:pmid/23086930#cont:162

AREG --+> MAPK3	Regulation	info:pmid/12743035#body:135
AREG --+> MAPK3	Regulation	info:pmid/23277062#cont:195
AREG --+> MAPK3	Regulation	info:pmid/21483252#cont:218
AREG --+> MAPK3	Regulation	info:pmid/20736313#cont:232
AREG --+> MAPK3	Regulation	info:pmid/21562239#cont:170
AREG --+> MAPK3	Regulation	info:pmid/21565184#body:140
AREG --+> MAPK3	Regulation	info:doi/10.1016/S0006-291X(03)00093-7#body:88
AREG --+> MAPK3	Regulation	info:doi/10.1053/j.gastro.2004.11.006#body:93
AREG --+> MAPK3	Regulation	info:doi/10.1186/1478-811X-11-86#cont:159
AREG --+> MAPK3	Regulation	info:doi/10.1016/j.mce.2011.12.005#body:48
AREG ---> chronic inflammation	Regulation	info:pmid/20498653#cont:327
AREG ---> immune response	Regulation	info:pmid/19494503#abs:1
KCTD13 ---> DNA repair	Regulation	info:pmid/23032261#cont:199
MAPK3 --+> Bipolar Disorder	Regulation	info:pmid/22884480#body:156
MAPK3 --+> chronic inflammation	Regulation	info:pmid/21983072#body:214
MAPK3 --+> chronic inflammation	Regulation	info:doi/10.1016/j.ajpath.2010.11.016#body:139
MAPK3 --+> mitochondrial depolarization	Regulation	info:pmid/15788397#abs:8
MAPK3 --+> mitochondrial depolarization	Regulation	info:pmid/12218054#abs:9
MAPK3 --+> mitochondrial depolarization	Regulation	info:pmid/16705147#abs:10
MAPK3 --+> mitochondrial depolarization	Regulation	info:pmid/20442495#cont:157
MAPK3 --+> mitochondrial depolarization	Regulation	info:pmid/18617697#body:221
MAPK3 --+> mitochondrial depolarization	Regulation	info:doi/10.1016/j.bpj.2012.11.3632#body:3
MAPK3 --+> neuronal death	Regulation	info:pmid/19843173#abs:5
MAPK3 --+> neuronal death	Regulation	info:pmid/10854274#abs:8
MAPK3 --+> neuronal death	Regulation	info:pmid/21933187#abs:7
MAPK3 --+> neuronal death	Regulation	info:pmid/21509715#cont:173
MAPK3 --+> neuronal death	Regulation	info:pmid/17664275#body:353
MAPK3 --+> neuronal death	Regulation	info:pmid/22094065#body:134
MAPK3 --+> neuronal death	Regulation	info:pmid/18635668#body:348
MAPK3 --+> neuronal death	Regulation	info:pmid/12676331#body:5
MAPK3 --+> neuronal death	Regulation	info:pmid/20971569#body:13
MAPK3 --+> neuronal death	Regulation	info:pmid/24055892#body:12
MAPK3 --+> neuronal death	Regulation	info:pmid/15893468#body:100
MAPK3 --+> neuronal death	Regulation	info:pmid/17870450#body:106
MAPK3 --+> neuronal death	Regulation	info:pmid/21422171#cont:462

MAPK3 --+> neuronal death	Regulation	info:pmid/17915333#body:203
MAPK3 --+> neuronal death	Regulation	info:pmid/17420100#body:11
MAPK3 --+> neuronal death	Regulation	info:pmid/21621864#body:97
MAPK3 --+> neuronal death	Regulation	info:pmid/23466131#body:14
MAPK3 --+> neuronal death	Regulation	info:pmid/24376709#cont:200
MAPK3 --+> neuronal death	Regulation	info:pmid/23439385#body:187
MAPK3 --+> neuronal death	Regulation	info:pmid/17888506#body:104
MAPK3 --+> neuronal death	Regulation	info:pmid/17716970#body:438
MAPK3 --+> neuronal death	Regulation	info:pmid/19201991#body:302
MAPK3 --+> neuronal death	Regulation	info:pmid/19766676#body:169
MAPK3 --+> neuronal death	Regulation	info:pmid/22275360#cont:172
MAPK3 --+> neuronal death	Regulation	info:pmid/17126142#body:97
MAPK3 --+> neuronal death	Regulation	info:pmid/24004478#cont:221
MAPK3 --+> neuronal death	Regulation	info:pmid/19799986#body:90
MAPK3 --+> neuronal death	Regulation	info:pmid/14769794#body:332
MAPK3 --+> neuronal death	Regulation	info:pmid/15193300#body:167
MAPK3 --+> neuronal death	Regulation	info:pmid/19293391#body:315
MAPK3 --+> neuronal death	Regulation	info:pmid/16537649#body:254
MAPK3 --+> neuronal death	Regulation	info:pmid/20463217#body:313
MAPK3 --+> neuronal death	Regulation	info:pmid/12392791#body:85
MAPK3 --+> neuronal death	Regulation	info:pmid/14552884#body:103
MAPK3 --+> neuronal death	Regulation	info:pmid/17524349#body:232
MAPK3 --+> neuronal death	Regulation	info:pmid/18354014#body:325
MAPK3 --+> neuronal death	Regulation	info:pmid/19038359#body:141
MAPK3 --+> neuronal death	Regulation	info:doi/10.1016/j.brainres.2010.05.079#body:94
MAPK3 --+> neuronal death	Regulation	info:doi/10.1016/j.brainres.2010.01.063#body:113
MAPK3 --+> neuronal death	Regulation	info:pmid/15153094#body:109
MAPK3 --+> synaptic transmission	Regulation	info:pmid/15086522#abs:2
MAPK3 --+> synaptogenesis	Regulation	info:pmid/18801922#body:95
MAPK3 --+> synaptogenesis	Regulation	info:pmid/20136499#cont:458
MAPK3 --+> synaptogenesis	Regulation	info:pmid/24157150#title:1
MAPK3 --+> synaptogenesis	Regulation	info:pmid/22394481#body:108
MAPK3 --+> synaptogenesis	Regulation	info:doi/10.1016/j.neuro.2012.02.013#body:108
MAPK3 ---> Autistic Disorder	Regulation	info:pmid/21848643#abs:12
MAPK3 ---> Autistic Disorder	Regulation	info:pmid/22555958#abs:6
MAPK3 ---> Autistic Disorder	Regulation	info:pmid/21595826#abs:13
MAPK3 ---> axon cargo transport	Regulation	info:pmid/12757748#body:408
MAPK3 ---> axonogenesis	Regulation	info:pmid/12367631#body:99
MAPK3 ---> axonogenesis	Regulation	info:pmid/23349896#cont:182
MAPK3 ---> axonogenesis	Regulation	info:doi/10.1016/j.mcn.2004.11.005#body:135

MAPK3 ---> brain development	Regulation	info:pmid/21248139#abs:11
MAPK3 ---> brain development	Regulation	info:pmid/22095091#abs:11
MAPK3 ---> DNA repair	Regulation	info:pmid/16052235#abs:6
MAPK3 ---> DNA repair	Regulation	info:pmid/21742020#body:135
MAPK3 ---> DNA repair	Regulation	info:pmid/19371614#body:250
MAPK3 ---> DNA repair	Regulation	info:pmid/23069143#body:116
MAPK3 ---> DNA repair	Regulation	info:pmid/11454682#body:210
MAPK3 ---> immune response	Regulation	info:pmid/19326394#abs:5
MAPK3 ---> immune response	Regulation	info:pmid/19091877#abs:7
MAPK3 ---> immune response	Regulation	info:pmid/19015047#body:76
MAPK3 ---> immune response	Regulation	info:pmid/22446503#body:109
MAPK3 ---> immune response	Regulation	info:pmid/9614082#body:253
MAPK3 ---> immune response	Regulation	info:pmid/21238948#body:159
MAPK3 ---> immune response	Regulation	info:pmid/18211830#body:117
MAPK3 ---> immune response	Regulation	info:pmid/16952481#body:151
MAPK3 ---> immune response	Regulation	info:pmid/23176885#body:8
MAPK3 ---> immune response	Regulation	info:pmid/21731758#cont:200
MAPK3 ---> immune response	Regulation	info:doi/10.1016/j.jss.2006.12.199#body:19
MAPK3 ---> immune response	Regulation	info:doi/10.1016/j.intimp.2012.03.005#body:109
MAPK3 ---> immune response	Regulation	info:pmid/18524942#body:53
MAPK3 ---> mitochondrial damage	Regulation	info:pmid/16045454#abs:9
MAPK3 ---> mitochondrial damage	Regulation	info:pmid/12218054#abs:6
MAPK3 ---> mitochondrial damage	Regulation	info:pmid/16167071#abs:6
MAPK3 ---> mitochondrial damage	Regulation	info:pmid/18594523#body:314
MAPK3 ---> mitochondrial damage	Regulation	info:pmid/20639456#body:138
MAPK3 ---> mitochondrial damage	Regulation	info:pmid/12507775#body:213
MAPK3 ---> mitochondrial damage	Regulation	info:pmid/15093752#body:70
MAPK3 ---> mitochondrial damage	Regulation	info:pmid/19563915#body:176
MAPK3 ---> mitochondrial damage	Regulation	info:pmid/23220614#body:94
MAPK3 ---> mitochondrial damage	Regulation	info:pmid/15905098#body:127
MAPK3 ---> mitochondrial damage	Regulation	info:pmid/23148948#body:15
MAPK3 ---> mitochondrial damage	Regulation	info:pmid/19919859#body:110

MAPK3 ---> mitochondrial damage	Regulation	info:doi/10.1016/S0006-291X(03)00060-3#body:125
MAPK3 ---> mitochondrial damage	Regulation	info:pmid/17079485#body:85
MAPK3 ---> mitochondrial damage	Regulation	info:pmid/14647418#body:119
MAPK3 ---> mitochondrial damage	Regulation	info:pmid/12492110#body:165
MAPK3 ---> nervous system development	Regulation	info:pmid/22095091#abs:1
MAPK3 ---> nervous system development	Regulation	info:pmid/18635547#body:165
MAPK3 ---> nervous system development	Regulation	info:pmid/22427945#cont:375
MAPK3 ---> nervous system development	Regulation	info:pmid/24411019#body:1
MAPK3 ---> nervous system development	Regulation	info:pmid/16880823#body:87
MAPK3 ---> Neurodegenerative Diseases	Regulation	info:pmid/21347512#cont:36
MAPK3 ---> Neurodegenerative Diseases	Regulation	info:pmid/24103374#body:94
MAPK3 ---> Neurodegenerative Diseases	Regulation	info:pmid/19038359#body:21
MAPK3 ---> Neurodegenerative Diseases	Regulation	info:pmid/18272143#body:14
MAPK3 ---> neuronal activity	Regulation	info:pmid/19541375#body:97
MAPK3 ---> neuronal activity	Regulation	info:pmid/17942733#body:54
MAPK3 ---> neuronal plasticity	Regulation	info:pmid/16326014#abs:1
MAPK3 ---> neuronal plasticity	Regulation	info:pmid/16431218#body:52
MAPK3 ---> neuronal plasticity	Regulation	info:pmid/17085074#body:17
MAPK3 ---> neuronal plasticity	Regulation	info:pmid/18272143#body:13
MAPK3 ---> neuronal plasticity	Regulation	info:pmid/18191237#body:99
MAPK3 ---> neuronal plasticity	Regulation	info:pmid/16139811#body:15
MAPK3 ---> neuronal plasticity	Regulation	info:pmid/20060019#body:202
MAPK3 ---> neuronal plasticity	Regulation	info:pmid/21167873#body:24
MAPK3 ---> neurotransmitter secretion	Regulation	info:pmid/15265865#body:61
MAPK3 ---> regulation of action potential	Regulation	info:pmid/17675293#body:83
MAPK3 ---> regulation of action potential	Regulation	info:pmid/12551961#body:220
MAPK3 ---| Nervous System Diseases	Regulation	info:pmid/20922651#cont:266
MAPK3 ---| synaptic vesicle exocytosis	Regulation	info:pmid/21430174#abs:3

MAPK3 ---| synaptic vesicle exocytosis	Regulation	info:pmid/23616533#cont:99
MTERFD1 --+> mitochondrial depolarization	Regulation	info:pmid/21381862#abs:10
MVP ---| DNA repair	Regulation	info:pmid/19251084#body:37
NAMPT --+> DNA repair	Regulation	info:pmid/20103674#body:312
NAMPT --+> MAPK3	Regulation	info:pmid/21945543#abs:7
NAMPT --+> MAPK3	Regulation	info:pmid/17408594#abs:5
NAMPT --+> MAPK3	Regulation	info:pmid/20647743#abs:3
NAMPT --+> MAPK3	Regulation	info:pmid/18952695#abs:5
NAMPT --+> MAPK3	Regulation	info:pmid/19906834#abs:9
NAMPT --+> MAPK3	Regulation	info:pmid/20375985#abs:8
NAMPT --+> MAPK3	Regulation	info:pmid/21327328#cont:103
NAMPT --+> MAPK3	Regulation	info:pmid/22089115#cont:328
NAMPT --+> MAPK3	Regulation	info:pmid/17543285#body:10
NAMPT --+> MAPK3	Regulation	info:pmid/23160967#cont:291
NAMPT --+> MAPK3	Regulation	info:pmid/21471274#cont:495
NAMPT --+> MAPK3	Regulation	info:pmid/23787158#body:148
NAMPT --+> MAPK3	Regulation	info:pmid/19081303#body:11
NAMPT --+> MAPK3	Regulation	info:pmid/22293189#body:57
NAMPT --+> MAPK3	Regulation	info:pmid/20537826#body:94
NAMPT --+> MAPK3	Regulation	info:pmid/21738955#cont:139
NAMPT --+> MAPK3	Regulation	info:pmid/19819277#body:85
NAMPT --+> MAPK3	Regulation	info:pmid/20920528#body:202
NAMPT --+> MAPK3	Regulation	info:pmid/23843684#cont:222
NAMPT --+> MAPK3	Regulation	info:pmid/23022179#body:302
NAMPT --+> MAPK3	Regulation	info:pmid/19393628#body:11
NAMPT --+> MAPK3	Regulation	info:doi/10.2174/138161212803582531#cont:650
NAMPT --+> MAPK3	Regulation	info:doi/10.2174/1871529X11313010007#cont:235
NAMPT ---> chronic inflammation	Regulation	info:pmid/19717018#body:7
NAMPT ---> DNA repair	Regulation	info:pmid/22704488#abs:5
NAMPT ---> immune response	Regulation	info:pmid/17374397#body:186
NAMPT ---> immune response	Regulation	info:pmid/23531116#cont:110
NAMPT ---> immune response	Regulation	info:pmid/20598369#body:13
NAMPT ---> immune response	Regulation	info:pmid/23466352#body:2
NAMPT ---> immune response	Regulation	info:pmid/19240195#body:130
NAMPT ---> immune response	Regulation	info:pmid/17507280#body:121
NAMPT ---> mitochondrial depolarization	Regulation	info:pmid/22044451#title:1
NAMPT ---> mitochondrial depolarization	Regulation	info:pmid/22960047#body:84

NAMPT ---> neuronal activity	Regulation	info:pmid/23071504#cont:102
NAMPT ---| mitochondrial damage	Regulation	info:pmid/21471274#cont:214
NAMPT ---| mitochondrial damage	Regulation	info:pmid/19661458#body:120
NAMPT ---| mitochondrial damage	Regulation	info:pmid/22904041#cont:309
NAMPT ---| neuronal death	Regulation	info:pmid/23843684#cont:295
NRXN1 --+> synaptic transmission	Regulation	info:pmid/19822762#body:64
NRXN1 --+> synaptic transmission	Regulation	info:pmid/23426688#cont:361
NRXN1 --+> synaptic transmission	Regulation	info:pmid/15858059#body:172
NRXN1 --+> synaptic transmission	Regulation	info:doi/10.1016/j.neuropharm.2011.01.024#body:127
NRXN1 ---> Asperger Syndrome	Regulation	info:pmid/19545994#abs:4
NRXN1 ---> Asperger Syndrome	Regulation	info:pmid/22798074#cont:236
NRXN1 ---> Autistic Disorder	Regulation	info:pmid/17034946#abs:7
NRXN1 ---> Autistic Disorder	Regulation	info:pmid/19545994#abs:4
NRXN1 ---> Autistic Disorder	Regulation	info:pmid/21477380#abs:1
NRXN1 ---> Autistic Disorder	Regulation	info:pmid/17498701#body:10
NRXN1 ---> Autistic Disorder	Regulation	info:pmid/22798074#cont:236
NRXN1 ---> Autistic Disorder	Regulation	info:pmid/18179900#body:62
NRXN1 ---> brain development	Regulation	info:pmid/21946175#body:128
NRXN1 ---> Child Development Disorders, Pervasive	Regulation	info:pmid/18179900#abs:7
NRXN1 ---> Mental Disorders	Regulation	info:doi/10.1016/j.neuropharm.2011.02.003#body:10
NRXN1 ---> neuron network morphology	Regulation	info:pmid/23536886#cont:280
NRXN1 ---> neurotransmitter secretion	Regulation	info:pmid/9430716#body:241
NRXN1 ---> neurotransmitter secretion	Regulation	info:pmid/15797875#body:67
NRXN1 ---> Schizophrenia	Regulation	info:pmid/21477380#abs:1
NRXN1 ---> Schizophrenia	Regulation	info:pmid/22348092#cont:14
NRXN1 ---> Schizophrenia	Regulation	info:pmid/21890328#body:91
NRXN1 ---> Schizophrenia	Regulation	info:pmid/21687627#cont:105
NRXN1 ---> Schizophrenia	Regulation	info:pmid/21946175#body:123
NRXN1 ---> synaptogenesis	Regulation	info:pmid/20064388#abs:7
NRXN1 ---> synaptogenesis	Regulation	info:pmid/18270208#abs:1
NRXN1 ---> synaptogenesis	Regulation	info:pmid/17868325#abs:2
NRXN1 ---> synaptogenesis	Regulation	info:pmid/15723836#body:170
NRXN1 ---> synaptogenesis	Regulation	info:pmid/21890328#body:5

NRXN1 ---> synaptogenesis	Regulation	info:pmid/17498701#title:1
NRXN1 ---> synaptogenesis	Regulation	info:pmid/20739565#cont:267
NRXN1 ---> synaptogenesis	Regulation	info:pmid/23133594#cont:366
NRXN1 ---> synaptogenesis	Regulation	info:pmid/18829439#body:192
NRXN1 ---> synaptogenesis	Regulation	info:pmid/21394644#cont:111
NRXN1 ---> synaptogenesis	Regulation	info:pmid/11173120#body:46
NRXN1 ---> synaptogenesis	Regulation	info:pmid/23358242#cont:21
NRXN1 ---> synaptogenesis	Regulation	info:pmid/21935445#cont:21
NRXN1 ---> synaptogenesis	Regulation	info:pmid/22617343#cont:27
NRXN1 ---> synaptogenesis	Regulation	info:pmid/19492049#body:120
NRXN1 ---> synaptogenesis	Regulation	info:doi/10.1016/j.ajhg.2013.02.007#body:16
PPP4C ---> DNA repair	Regulation	info:pmid/21135129#abs:7
PTDSS1 --+> axonogenesis	Regulation	info:pmid/18343815#body:320
RYR2 --+> mitochondrial depolarization	Regulation	info:pmid/18950706#body:83
RYR2 --+> neurotransmitter secretion	Regulation	info:pmid/21113126#body:191
RYR2 --+> regulation of action potential	Regulation	info:pmid/24141185#cont:228
RYR2 --+> regulation of action potential	Regulation	info:pmid/12543086#body:251
RYR2 --+> regulation of action potential	Regulation	info:pmid/22723297#cont:379
RYR2 ---> Developmental Disabilities	Regulation	info:pmid/22814392#abs:8
RYR2 ---> nervous system development	Regulation	info:pmid/18602130#abs:7
RYR2 ---> synaptic transmission	Regulation	info:pmid/19641109#body:170
SPN ---> immune response	Regulation	info:pmid/12055210#abs:6
SPN ---> immune response	Regulation	info:pmid/10908570#body:316
SPN ---> immune response	Regulation	info:pmid/11024037#body:65
SPN ---> immune response	Regulation	info:pmid/9351819#body:211
SPN ---> immune response	Regulation	info:pmid/16246302#body:11
SPN ---> immune response	Regulation	info:pmid/21768350#cont:31
SPN ---> immune response	Regulation	info:pmid/16890924#body:15
SPN ---> immune response	Regulation	info:pmid/10521289#body:123
SPN ---> immune response	Regulation	info:doi/10.1016/S0966-842X(00)01837-0#body:7
SPN ---> immune response	Regulation	info:pmid/9862667#body:48
SPN ---> immune response	Regulation	info:pmid/10521289#body:569
SPN ---> immune response	Regulation	info:pmid/15606794#body:394
SPN ---| synaptogenesis	Regulation	info:pmid/19351959#body:105
TAOK2 ---> neuronal activity	Regulation	info:pmid/19541375#body:97
TBX6 ---> neuronal plasticity	Regulation	info:pmid/19070637#body:248

<table>
<tr><td></td><td></td><td></td></tr>
<tr><td>TBX6 ---| nervous system development</td><td>Regulation</td><td>info:pmid/10468588#body:46</td></tr>
<tr><td>ZIC1 ---> Developmental Disabilities</td><td>Regulation</td><td>info:pmid/23313878#body:79</td></tr>
<tr><td>ZIC1 ---> MAPK3</td><td>Regulation</td><td>info:pmid/22799764#cont:86</td></tr>
<tr><td>ZIC1 ---> MAPK3</td><td>Regulation</td><td>info:pmid/21347233#cont:55</td></tr>
<tr><td>ZIC1 ---> nervous system development</td><td>Regulation</td><td>info:pmid/22799764#cont:21</td></tr>
<tr><td>ZIC1 ---| ZIC4</td><td>Expression</td><td>info:pmid/15210192#body:101</td></tr>
<tr><td>ZIC4 ---> Developmental Disabilities</td><td>Regulation</td><td>info:pmid/23313878#body:79</td></tr>
<tr><td>ZIC4 ---> nervous system development</td><td>Regulation</td><td>info:pmid/15895369#body:170</td></tr>
<tr><td>ZIC4 ---| ZIC1</td><td>Expression</td><td>info:pmid/15210192#body:101</td></tr>
<tr><td>ZP4 --+> immune response</td><td>Regulation</td><td>info:pmid/22415139#abs:11</td></tr>
</table>

Supplementary table 5. General information about the families included in the study.

Family ID	Consanguineous	Family fully analyzed	Family member ID	Status	Gender	Diagnosis
SES 2	X	X	F2-05	Autistic	M	Autistic
			F2-06	Sibling	F	Non autistic
			F2-07	Sibling	M	Non autistic
			F2-08	Father	M	Non autistic
			F2-09	Mother	F	Non autistic
SES 3		X	F3-10	autistic child	M	Autistic
			F3-11	autistic child	F	Autistic
			F3-12	Sibling	M	Non autistic
			F3-13	Father	M	Non autistic
			F3-14	Mother	F	Non autistic
SES 5		X	F5-20	autistic child	M	Autistic
			F5-21	Sibling	M	Non autistic
			F5-22	Father	M	Non autistic
			F5-23	Mother	F	Non autistic
LAS 6		X	F6-24	autistic child	M	Autistic
			F6-26	Sibling	F	Non autistic
			F6-27	Father	M	Non autistic
			F6-28	Mother	F	Non autistic
LAS 7		X	F7-29	autistic child	M	Autistic
			F7-30	Sibling	F	Non autistic

			F7-31	Sibling	F	Non autistic
			F7-32	Father	M	Non autistic
			F7-33	Mother	F	Non autistic
LAS 8		x	F8-34	autistic child	M	Autistic
			F8-36	Father	M	Non autistic
			F8-37	Mother	F	Non autistic
LAS 9		x	F9-38	autistic child	M	Autistic
			F9-39	Sibling	M	Non autistic
			F9-40	Father	M	Non autistic
			F9-41	Mother	F	Non autistic
LAS 10	x		F10-42	autistic child	M	Autistic
			F10-43	Sibling	M	Non autistic
			F10-44	Sibling	M	Non autistic
			F10-45	Sibling	M	Non autistic
			F10-47	Mother	F	Non autistic
LAS 11	x	x	F11-48	autistic child	M	Autistic
			F11-49	Sibling	F	Non autistic
			F11-50	Sibling	F	Non autistic
			F11-51	Father	M	Non autistic
			F11-52	Mother	F	Non autistic
LAS 15	x	x	F15-70	autistic child	M	Autistic
			F15-71	Sibling	F	Non autistic
			F15-72	Sibling	M	Non autistic
			F15-73	Father	M	Non autistic
			F15-74	Mother	F	Non autistic
LAS 17		x	F17-80	autistic child	M	Autistic
			F17-81	Father	M	Non autistic
			F17-82	Mother	F	Non autistic
CLIN 18		x	F18-83	autistic child	M	Autistic
			F18-84	Sibling	M	Non autistic
			F18-86	Father	M	Non autistic
			F18-87	Mother	F	Non autistic
CLIN 19		x	F19-88	autistic child	M	Autistic
			F19-89	autistic child	M	Autistic
			F19-90	Sibling	F	Non autistic
			F19-91	Father	M	Non autistic
			F19-92	Mother	F	Non autistic
CLIN 20		x	F20-93	autistic child	M	Autistic
			F20-94	Sibling	F	Non autistic
			F20-95	Sibling	F	Non autistic

			F20-96	Father	M	Non autistic
			F20-97	Mother	F	Non autistic
CLIN22		x	F22-101	autistic child	M	Autistic
			F22-102	Father	M	Non autistic
			F22-103	Mother	M	Non autistic
CLIN24		x	F24-108	autistic child	M	Autistic
			F24-109	Sibling	F	Non autistic
			F24-110	Sibling	M	Non autistic
			F24-111	Father	M	Non autistic
			F24-112	Mother	F	Non autistic
CLIN27	x	x	F27-122	autistic child	M	Autistic
			F27-123	Sibling	F	Non autistic
			F27-124	Sibling	M	Non autistic
			F27-125	Father	M	Non autistic
			F27-126	Mother	F	Non autistic
CLIN28	x	x	F28-127	autistic child	M	Autistic
			F28-131	Father	M	Non autistic
			F28-132	Mother	F	Non autistic
CLIN29		x	F29-133	autistic child	M	Autistic
			F29-134	Sibling	F	Non autistic
			F29-136	Father	M	Non autistic
			F29-137	Mother	F	Non autistic
SAI31		x	F31-145	autistic child	M	Autistic
			F31-146	Father	M	Non autistic
			F31-147	Mother	F	Non autistic
SAI32	x	x	F32-148	Autistic	M	Autistic
			F32-149	Sibling	M	Non autistic
			F32-150	Father	M	Non autistic
			F32-151	Mother	F	Non autistic
SAI33		x	F33-152	autistic child	M	Autistic
			F33-156	Father	M	Non autistic
			F33-157	Mother	F	Non autistic
SAI34		x	F34-158	autistic child	M	Autistic
			F34-159	Sibling	F	Non autistic
			F34-160	Sibling	M	Non autistic
			F34-161	Sibling	F	Non autistic
			F34-162	Father	M	Non autistic
			F34-163	Mother	F	Non autistic
CLIN35		x	F35-164	autistic child	F	Autistic
			F35-165	Sibling	F	Non autistic

			F35-166	Sibling	F	Non autistic
			F35-167	Father	M	Non autistic
			F35-168	Mother	F	Non autistic
CLIN36		x	F36-169	autistic child	M	Autistic
			F36-170	Father	M	Non autistic
			F36-171	Mother	F	Non autistic
BAK37		x	F37-172	autistic child	M	Autistic
			F37-173	Sibling	F	Non autistic
			F37-174	Father	M	Non autistic
			F37-175	Mother	F	Non autistic
BAK38		x	F38-176	autistic child	M	Autistic
			F38-177	Sibling	M	Non autistic
			F38-178	Father	M	Non autistic
			F38-179	Mother	F	Non autistic
BAK39		x	F39-180	autistic child	M	Autistic
			F39-181	Sibling	F	Non autistic
			F39-182	Sibling	M	Non autistic
			F39-184	Father	M	Non autistic
			F39-185	Mother	F	Non autistic
BAK40		x	F40-186	autistic child	M	Autistic
			F40-187	Sibling	F	Non autistic
			F40-188	Father	M	Non autistic
			F40-189	Mother	F	Non autistic
BAK41		x	F41-190	autistic child	M	Autistic
			F41-192	Father	M	Non autistic
			F41-193	Mother	F	Non autistic
BAK42		x	F42-194	autistic child	M	Autistic
			F42-195	Father	M	Non autistic
			F42-196	Mother	F	Non autistic
CLIN43		x	F43-197	autistic child	M	Autistic
			F43-198	Sibling	F	Non autistic
			F43-199	Father	M	Non autistic
			F43-200	Mother	F	Non autistic
CLIN44		x	F44-201	autistic child	M	Autistic
			F44-202	Sibling	m	Non autistic
			F44-203	Father	M	Non autistic
			F44-204	Mother	F	Non autistic
CLIN45	x	x	F45-205	autistic child	M	Autistic
			F45-206	Sibling	F	Non autistic
			F45-207	Sibling	M	Non autistic

			F45-208	Sibling	M	Non autistic
			F45-209	Father	M	Non autistic
			F45-210	Mother	F	Non-autistic
CLIN46			F46-211	Sibling	F	Autistic
		x	F46-212	Father	M	Non autistic
			F46-213	Mother	F	Non autistic
Clin47			F47-214	Sibling	F	Autistic
			F47-215	Sibling	M	Non autistic
		x	F47-216	Father	M	Non autistic
			F47-217	Mother	F	Non autistic
1A68			1A68-	autistic child	M	Autistic
1A69			1A69-	autistic child	M	Autistic
3A146			3A146-	autistic child	M	Autistic

Supplementary tables legends

Supplementary table 1. *De novo* CNVs identified in patients with ASD classified as benign and likely benign. The chromosome location, as well as the CNV type (gain referring to a duplication, loss referring to a deletion), length and gene content are given. Genomic positions are given according to Human Genome Building GCRh37, HG19.

Supplementary table 2. Inherited CNVs identified in patients with ASD classified as benign and likely benign. The chromosome location, as well as the CNV type (gain referring to a duplication, loss referring to a deletion), length and gene content are given. Genomic positions are given according to Human Genome Building GCRh37, HG19.

Supplementary table 3. Gene ontology (GO) enrichment analysis among genes included in all CNVs classified as pathogenic, likely pathogenic, and of unknown significance. Only categories reaching a statistical significance of Fisher Exact P-value corrected for multiple comparisons in Benjamini-Hochberg test are shown. Significance was set to a P-value <0.05.

Supplementary table 4. Studies and citations for each relation within the network build between genes included in CNVs classified as pathogenic, likely pathogenic, and of unknown significance and different entities related to identified deficits in ASDs using Pathway studio.

Supplementary table 5. General information about the families included in the study.
